# Laser speckle flowgraph reveals dynamic characteristics and clinical relevance of choroidal watershed and peripapillary hypoperfusion zones

**DOI:** 10.1038/s41598-026-47062-z

**Published:** 2026-04-04

**Authors:** Ryuya Hashimoto, Randy H. Kardon

**Affiliations:** 1 Department of Ophthalmology and Visual Sciences, University of Iowa, Highway 6, Iowa City, IA 52242 USA; 2 Iowa City VA Center for the Prevention and Treatment of Visual Loss, Iowa City, IA USA

**Keywords:** Choroidal watershed zone, Peripapillary choroid, Ocular blood flow, Pulse waveform, Pulsatility, Laser speckle flowgraphy, Biomarkers, Diseases, Medical research

## Abstract

The hemodynamic characteristics of the choroidal watershed zone (CWZ) have not been fully quantified. We aimed to characterize the blood flow and pulsatility within the choroidal hypoperfusion zone (CHZ) and investigate its relationship with adjacent optic nerve head (ONH) perfusion in healthy eyes, using laser speckle flowgraphy (LSFG). Blood flow and pulsatile waveform metrics were compared within and outside the CHZ and in optic nerve head (ONH) sectors in 100 healthy participants. Two patients with non-arteritic anterior ischemic optic neuropathy (NAION) were also reviewed as the illustrative cohort. In the illustrative cohort, the CHZ in LSFG color maps showed qualitative spatial correlation to areas of delayed choroidal filling on fluorescein angiography. In the main cohort, average flow was lower by 44.3 ± 15.4% and the average, minimum, and maximum blood flow and pulsatility measures were significantly lower (*P* < 0.0001 for key indices) within the CHZ than outside. Time to peak systolic blood flow was delayed (*P* = 0.028). The temporal-to-nasal flow ratio in the ONH was significantly reduced when the CHZ was more temporally located relative to the optic nerve center (*P* = 0.0015). In conclusion, the tissue located within the CWZ is more vulnerable to drops in ocular perfusion pressure.

**Supplementary Information:**

The online version contains supplementary material available at 10.1038/s41598-026-47062-z.

## Introduction

When the choroid is supplied by two or more arteries, the boundary between the territories of any two end-arteries is called a watershed zone^1^. Hayreh first identified the choroidal watershed zone (CWZ) using fluorescein angiography (FA) and demonstrated its significance: during episodes of reduced ocular perfusion pressure (OPP) in the ocular vascular beds supplied by one or more arteries, the CWZ is considered particularly vulnerable to ischemia^1^. Additionally, the CWZ between various posterior ciliary arteries (PCAs) plays an essential role in imparting additional risk of vision loss when the optic nerve head (ONH) is located within it, in ischemic disorders, including glaucoma and non-arteritic anterior ischemic optic neuropathy (NAION)^2^. The portion of the ONH located within the CWZ is more susceptible to ischemia than other areas^2^. Hayreh previously demonstrated that the peripapillary choroidal hypoperfusion zone, which is a part of the CWZ, is the most vulnerable region during hypotension or raised intraocular pressure compared to other choroidal areas. The location and spatial distribution of the CWZ varies between eyes, depending upon the anatomical location of the end arteries, which is determined during development, the arterial resistance of these end arteries and the ocular perfusion pressure. The main CWZ zone in most eyes lies between the margin of the disc and the point where the PCAs enter the eyeball^3^. Many previous studies have demonstrated a substantial clinical relevance between the CWZ and the progression of diseases such as NAION^2^, glaucoma^4^, and age-related macular degeneration^5–8^. Therefore, it is essential to investigate and clarify the features and blood flow pulsatility in the choroidal watershed and peripapillary choroidal hypoperfusion zone (CHZ).

Previously, in order to assess the choroidal watershed and peripapillary hypoperfusion zones, intravenous fluorescent dye injection utilizing FA or indocyanine green (ICG) angiography has been used to identify areas of delayed choroidal filling. Giuffre et al.^9^ reported that a well-outlined CWZ was observed using FA in 44.6% of 800 normal participants. While ICG angiography offers enhanced visualization of the CWZ^10^, detecting a well-defined CWZ can still be challenging with both digital FA and ICG angiography. This is often because of technical difficulties such as slower digital sampling rate of the higher pixel density retina imaging devices currently used, making it more difficult to capture the early phase of filling of the choroid with dye. Furthermore, because of the invasive nature of these procedures, they are not performed routinely.

Laser speckle flowgraphy (LSFG-NAVI^®^, Softcare Co. Ltd., Fukuoka, Japan) is a noninvasive quantitative method for measuring dynamic blood flow during the cardiac cycle in the ONH, retina, and choroid. This technique is based on the changes in the contrast of the speckle pattern of laser light reflected from the fundus of the eye, which is inversely proportional to the movement speed of red blood cells at each image pixel, quantified specifically by the mean blur rate (MBR)^11,12^. The LSFG technique has been successfully employed to evaluate ocular blood flow and autoregulatory capacity in the ONH^13–16^ and retinal artery^17–19^. In addition to these ocular vascular beds, LSFG has advantages in the evaluation of choroidal blood flow and pulsatile flow waveforms in humans^20–26^. LSFG has also been used to study ONH and choroidal blood flow in aging macular degeneration (AMD) and ischemic optic neuropathy^27,28^.

We hypothesized that the portion of the ONH located within the CWZ may exhibit decreased blood flow making it more susceptible to ischemia from drops in ocular perfusion pressure. However, the hemodynamic characteristics within the CWZ have not been quantitatively examined in humans. This study therefore aimed to: (1) qualitatively assess the correspondence between LSFG patterns and FA findings in illustrative clinical cases; (2) characterize the blood flow and pulsatility within the CHZ versus outside the CHZ in healthy eyes using LSFG; and (3) investigate the relationship between the spatial location of the CHZ and sectoral blood flow in the ONH.

## Results

### Comparison of the choroidal hypoperfusion zone between fluorescein angiography and panoramic LSFG color coded blood flow map

**Fig. 1:**
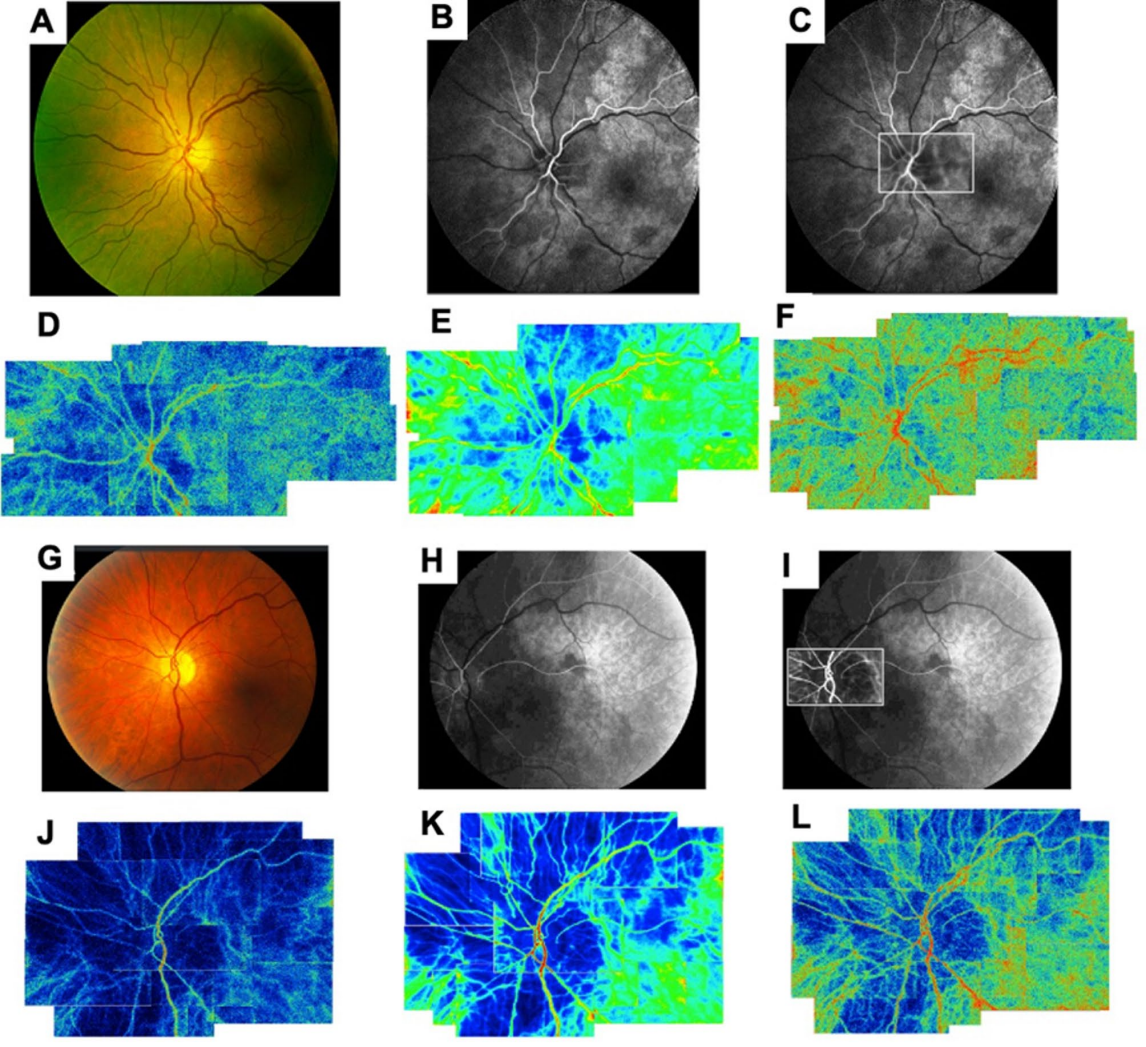
Representative images of Panoramic laser speckle flowgraphy map and fluorescein angiography in eyes with acute and chronic non-arteritic ischemic optic neuropathy. Panoramic laser speckle flowgraphy (LSFG) and fluorescein angiography (FA) showing the choroidal hypoperfusion zone (CHZ) and its cardiac-phase dynamics in acute and chronic NAION. Acute NAION (A–F; 72-year-old woman, BCVA 20/40; Goldmann visual field: inferior altitudinal defect) (**a**) Fundus photographs obtained at initial visit. The left eye showed minimal optic disc edema and peripapillary hemorrhage. (**b**) Fluorescein angiography performed in the early phase. The choroidal hypoperfusion zone (CHZ) was located in the superior, temporal, and inferior nasal regions. (**c**) To show the spatial correspondence, the monochrome LSFG flow map (small rectangular area located at the optic nerve head) is overlaid on the FA image. The area of correspondence is highlighted with a white outline for clarity. (**d**) Panoramic LSFG color map, composed of a blood flow map during the diastolic phase, displays a wider CHZ. (**e**) Panoramic LSFG color map, based on a composite map (average blood flow map over entire cardiac cycle), shows the CHZ in the superior, temporal, and nasal regions. (**f**) Panoramic LSFG color map, derived from a blood flow map during the systolic phase, reveals a narrower CHZ with sufficient blood flow. Chronic NAION (G–L; 63-year-old man, BCVA 20/50; Goldmann visual field: central scotoma with superimposed inferior defect), (**g**) Fundus photographs obtained at initial visit. The left eye showed no disc edema, and pallor was most marked temporally. (**h**) Fluorescein angiography performed in the early phase. This figure shows a vertical choroidal hypoperfusion zone (CHZ) located mainly in the temporal region. (**i**) The overlaid monochrome LSFG map corresponds to the area of delayed filling on FA. The area of correspondence is highlighted with a white outline for clarity. (**j**) Panoramic LSFG color map, composed of a blood flow map during the diastolic phase, shows wider CHZ with insufficient blood flow. (**k**) Panoramic LSFG color map composed of a composite map, with the optic nerve head positioned at the center of the CHZ. (**l**) Panoramic LSFG color map, derived from a blood flow map during the systolic phase, shows a narrower CHZ with sufficient blood flow.

In two illustrative patients with acute (a 72-year-old woman, visual acuity 20/40) and chronic (a 63-year-old man, visual acuity 20/50) NAION who had FA performed, a panoramic LSFG color blood flow map distinctly delineated the CHZ, exhibiting qualitative correspondence to the spatial distribution of the CWZ obtained from the early filling phase of fluorescein angiography in the same eye (Fig. 1).

To visualize the spatial correlation between choroidal hypoperfusion and LSFG-derived blood flow patterns, Fig. 1c and i represent composite images in which a monochrome LSFG blood flow map centered on the ONH was overlaid onto the early-phase FA shown in Fig. 1b and h. Additionally, the panoramic LSFG color map illustrated the CHZ during the trough of the diastolic phase, the average of all time points during the cardiac cycle, and the peak systolic phase, revealing an expansion of the CHZ during the diastolic phase in both patients with NAION (Fig. 1d and j). Expansion of the CHZ during diastole was present in every eye and demonstrated the dynamic characteristics of the spatial distribution of choroidal hypoperfusion. Blood flow was always lowest in the center of the CHZ, and the area of hypoperfusion relative to the surrounding choroid becomes greater when OPP became less during diastole.

### Clinical characteristics of the 100 healthy eyes

The clinical characteristics of the 100 healthy participants (57 females and 43 males) are shown in Table 1. The mean age was 42.2 ± 14.7 years. No significant differences were observed in age, body mass index, heart rate, or IOP between the male and female groups. In contrast, systolic, diastolic and mean arterial blood pressures and ocular perfusion pressures were significantly higher in the male group than in the female group (e.g., mean OPP: 50.4 ± 7.47 vs. 45.9 ± 5.49 mmHg, *P* = 0.001).

**Table 1:** Clinical Characteristics of study participants.

	Overall	Male	Female	*P*-value
	(*n* = 100)	(*n* = 43)	(*n* = 57)	
Subjects (n)	100	43	57	
Age (years)	42.2 ± 14.7	40.4 ± 15.0	43.5 ± 14.5	0.251
Body mass Index (kg/m^2^)	28.5 ± 6.82	28.1 ± 4.68	28.8 ± 8.10	0.764
Heart rate (bpm)	70.0 ± 12.0	68.6 ± 11.8	71.1 ± 12.2	0.334
Intraocular pressure (mmHg)	14.4 ± 2.87	14.0 ± 2.75	14.8 ± 2.93	0.268
Systemic blood Pressure (mmHg)	127 ± 13.5	130 ± 10.7	125 ± 15.0	0.024
Diastolic blood pressure (mmHg)	76.5 ± 9.93	79.6 ± 9.79	74.1 ± 9.43	0.006
Mean blood pressure (mmHg)	93.4 ± 10.0	96.5 ± 8.78	91.0 ± 10.3	0.006
Ocular perfusion pressure (mmHg)	47.8 ± 7.02	50.4 ± 5.49	45.9 ± 7.47	0.001

Data are expressed as the mean ± standard deviation.

*P*-values were obtained from Mann-Whitney test.

BMI, body mass index; IOP, Intraocular pressure; SBP, systolic blood pressure; DBP, diastolic blood pressure; MBP, Mean blood pressure; OPP, ocular perfusion pressure.

### Visual classification of choroidal hypoperfusion zone symmetry and asymmetry patterns using LSFG color coded blood flow maps

Based on qualitative assessment of choroidal blood flow distribution patterns surrounding the optic nerve head on LSFG color coded blood flow map, eyes were categorized into two groups: the asymmetry group and the symmetry group. Among the 100 healthy eyes evaluated, 67 eyes (67%) were classified as asymmetrical (temporal optic nerve head lies within the CHZ) and 33 eyes (33%) as symmetrical (entire optic nerve head lies within the CHZ). The precise operational criteria for classification were as follows: Asymmetric pattern — the CHZ on the average LSFG color map is confined to the temporal quadrant relative to the ONH center; the temporal ONH margin lies within or at the boundary of the CHZ, while the nasal ONH margin lies outside it. Symmetric pattern — the CHZ encircles the entire ONH on the average LSFG color map, such that both the temporal and nasal ONH margins lie within or at the boundary of the CHZ. Classification was performed by a single trained examiner (R.H.) without independent second-observer verification; formal inter-rater reliability assessment was not conducted, which is a limitation of this study.

**Fig. 2:**
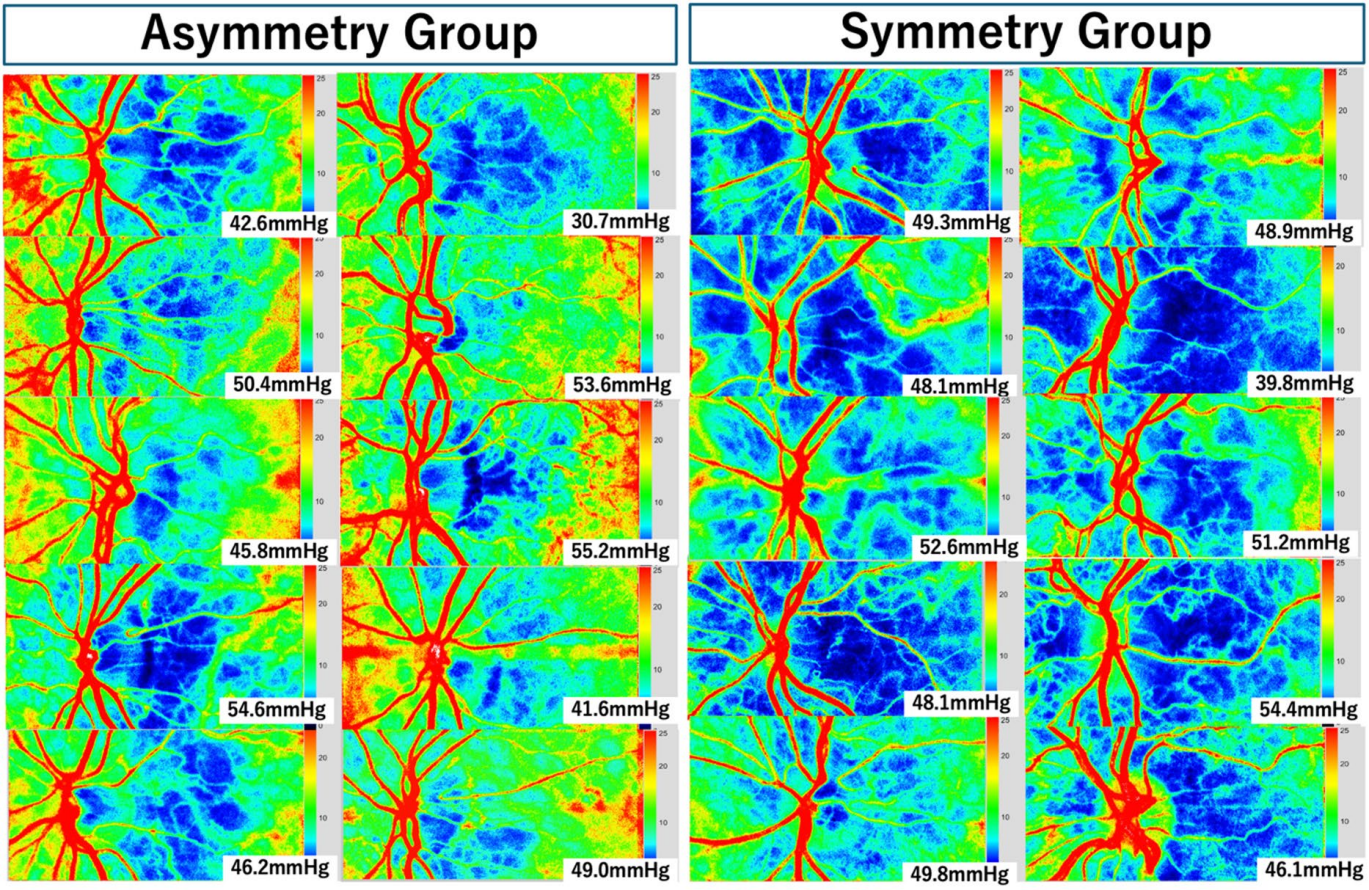
Representative laser speckle flowgraphy map images showing choroidal blood flow symmetry and asymmetry patterns. Representative LSFG color-coded blood flow composite maps from 20 eyes are shown. Based on spatial configuration of the choroidal perfusion zone surrounding the optic nerve head, eyes were categorized into Asymmetry group (left panel, n = 10) or the Symmetry group (right panel, n = 10). The ocular perfusion pressure is shown in the lower right corner of each panel. Blue areas correspond to regions with reduced mean blur rate, indicating lower choroidal blood flow. The Symmetry group exhibited a broader extent of low-perfusion areas despite comparable OPP values between the two groups.

Ten representative eyes from each group (*n* = 10 per group) were selected as examples and are shown in Fig. 2. Each panel displays the OPP in the sitting position, calculated as 2/3 mean MBP minus IOP, as defined in the Methods. While no significant difference in OPP was observed between the asymmetry and symmetry groups, the extent of low-perfusion regions, represented by blue areas indicating reduced MBR on LSFG color coded blood flow maps, was greater in the symmetry group than in the asymmetry group. This finding suggests that the spatial pattern of choroidal perfusion may be influenced not only by OPP, but also by the distribution of choroidal end-arterioles and their vascular resistance.

### Comparison of LSFG parameters and vascular resistance between the choroidal hypoperfusion zone and outside the hypoperfusion zone

Average MBRs within the CHZ were reduced to 44.3 ± 15.4% of blood flow in the adjacent choroid outside of the CHZ. All primary flow metrics were significantly lower within the CHZ compared with the adjacent region (Wilcoxon signed-rank test): average MBR (4.21 ± 1.87 vs. 9.32 ± 1.32 AU), maximum MBR (5.44 ± 2.36 vs. 11.90 ± 1.76 AU), and minimum MBR (3.21 ± 1.47 vs. 7.23 ± 1.16 AU; all *P* < 0.0001). Vascular resistance (OPP/MBR) was significantly higher within the CHZ (13.50 ± 6.16 vs. 5.13 ± 1.02 mmHg/AU, *P* < 0.0001). Pulse-waveform analysis also revealed significant differences: Beat Strength (3.93 ± 1.76 vs. 8.03 ± 2.13), Flow Acceleration Index (FAI; 0.701 ± 0.358 vs. 1.47 ± 0.417), and Blowout Score (BOS; 72.1 ± 6.21 vs. 74.0 ± 5.72) were all significantly lower within the CHZ (all *P* < 0.0001). Conversely, the Acceleration Time Index (ATI) was longer (27.9 ± 5.31 vs. 27.1 ± 5.35, *P* = 0.028) and the Resistive Index (RI) was higher (0.410 ± 0.070 vs. 0.388 ± 0.070, *P* < 0.0001) inside the CHZ. No significant inter-regional differences were observed for Blowout Time (BOT; 47.7 ± 5.00 vs. 48.0 ± 5.52, *P* = 0.209) or skew (13.6 ± 2.14 vs. 13.3 ± 2.14, *P* = 0.078).

### Comparison of LSFG parameters and vascular resistance within and outside the choroidal hypoperfusion zone between eyes with asymmetric and symmetric patterns

Table 2 summarizes the comparison of LSFG parameters between the asymmetry group (*n* = 67; 27 males and 40 females) and symmetry group (*n* = 33; 16 males and 17 females), both within and outside the CHZ. There was no significant difference in sex distribution between the two groups (Fisher’s exact test, *P* = 0.5208). Within the CHZ, ATI was significantly higher in the symmetry group (29.8 ± 0.81 vs. 27.0 ± 0.66, *P* = 0.009), while skew was significantly lower (13.1 ± 0.37 vs. 13.9 ± 0.26, *P* = 0.048). Outside the CHZ, these trends persisted: ATI remained higher (29.4 ± 0.89 vs. 26.0 ± 0.63, *P* = 0.005), and skew lower (12.5 ± 0.42 vs. 13.7 ± 0.23, *P* = 0.012) in the symmetry group. A significant difference in FAI was also noted outside the CHZ (1.35 ± 0.46 vs. 1.53 ± 0.38, *P* = 0.045), though not within. No significant differences were found in MBR (average, max, min), vascular resistance, beat strength, RI, BOS, or BOT in either region.

**Table 2:** Comparison of LSFG parameters and vascular resistance between asymmetry and symmetry groups within and outside the choroidal hypoperfusion zone.

	CHZ in the Asymmetry Group (*n* = 67)	CHZ in the Symmetry Group (*n* = 33)	*P*-value	Outside the CHZ in the Asymmetry Group (*n* = 67)	Outside the CHZ in the Symmetry Group (*n* = 33)	*P*-value
Average MBR (AU)	4.21 ± 1.68	4.20 ± 2.24	0.489	9.32 ± 1.15	9.32 ± 1.64	0.814
Maximum MBR (AU)	5.48 ± 2.13	5.37 ± 2.80	0.372	11.9 ± 1.49	11.8 ± 2.22	0.611
Minimum MBR (AU)	3.19 ± 1.31	3.26 ± 1.76	0.690	7.18 ± 1.04	7.34 ± 1.39	0.842
Vascular Resistance	12.9 ± 4.93	14.7 ± 8.08	0.599	5.21 ± 0.99	5.26 ± 1.10	0.574
Beat Strength	4.07 ± 1.71	3.66 ± 1.90	0.118	8.24 ± 1.90	7.60 ± 2.51	0.120
FAI	0.73 ± 0.34	0.65 ± 0.39	0.147	1.53 ± 0.38	1.35 ± 0.46	0.045
ATI	27.0 ± 0.66	29.8 ± 0.81	0.009	26.0 ± 0.63	29.4 ± 0.89	0.005
RI	0.42 ± 0.07	0.40 ± 0.08	0.062	0.40 ± 0.07	0.37 ± 0.07	0.222
BOS	56.2 ± 5.70	62.4 ± 7.04	0.108	75.1 ± 5.67	84.8 ± 5.70	0.306
BOT	33.6 ± 4.78	37.3 ± 5.40	0.204	48.3 ± 5.53	48.5 ± 5.49	0.292
Skew	13.9 ± 0.26	13.1 ± 0.37	0.048	13.7 ± 0.23	12.5 ± 0.42	0.012

*P*-values were obtained from the Mann-Whitney U test.

CHZ, Choroidal hypoperfusion zone; MBR, Mean blur rate; Beat strength, BS; FAI, Flow acceleration index; ATI. Acceleration time index; RI, Resistance index; BOS, Blowout score; BOT, Blowout time.

### Representative pulsatile waveform images of laser speckle flowgraphy in the choroidal hypoperfusion zone and outside the hypoperfusion zone

**Fig. 3:**
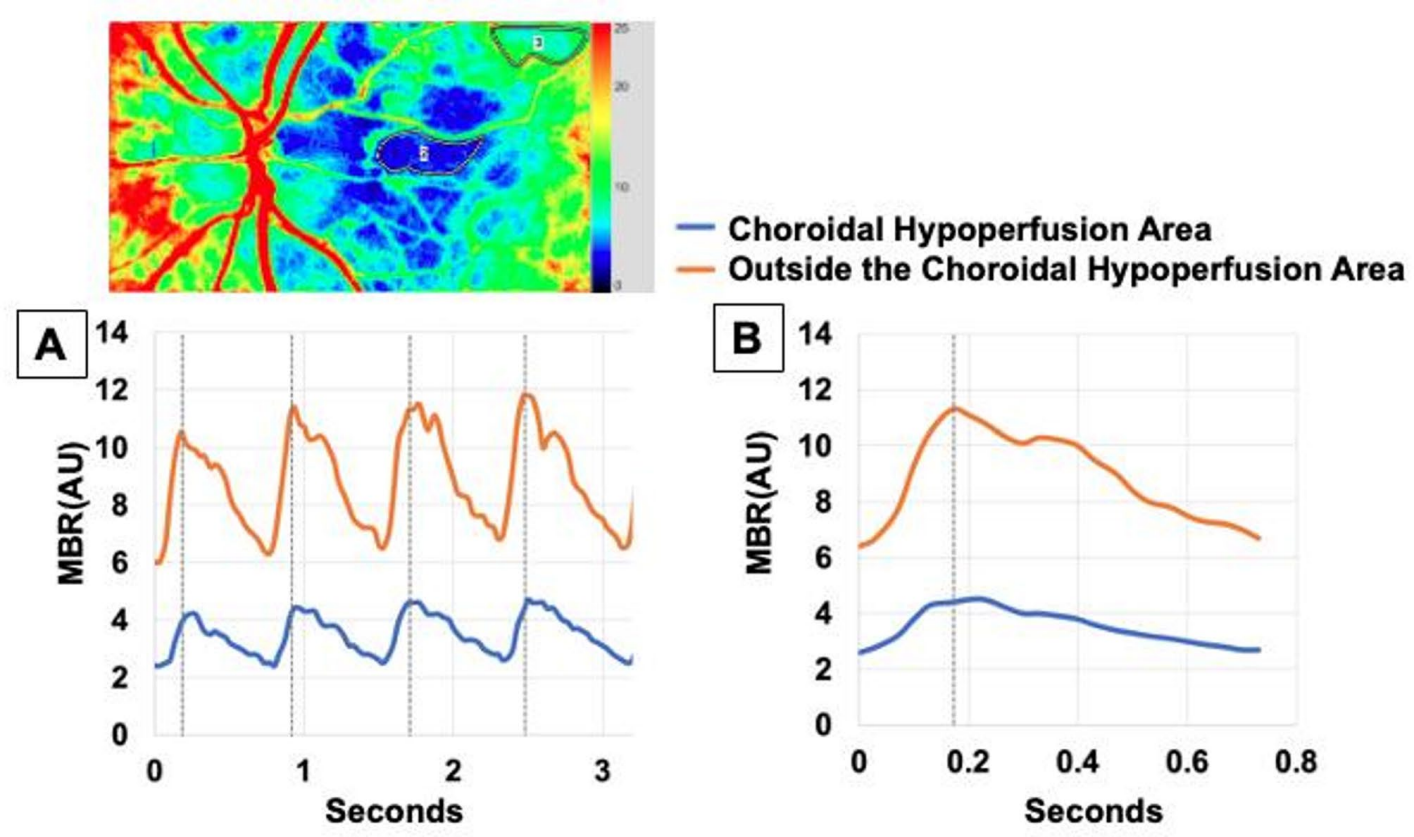
Comparison of Pulse Waveforms between the Choroidal Hypoperfusion Zone (CHZ) and a region outside the CHZ in a healthy eye. The LSFG map (top panel) shows two representative regions of interest (ROIs) for analysis: one placed within the CHZ (ROI 2) and one in an adjacent, well-perfused area outside the CHZ (ROI 3). The graphs below display the MBR waveforms extracted from these corresponding ROIs. As indicated by the legend, the blue line represents the waveform from within the CHZ, and the orange line represents the waveform from outside the CHZ. (**a**) MBR waveforms over four consecutive cardiac cycles. (**b**) An averaged single cardiac cycle waveform. The waveform from outside the CHZ (orange line) demonstrates a higher average MBR, greater pulsatility (amplitude), and a shorter time to peak MBR compared to the waveform from within the CHZ (blue line).

Figure 3 illustrates the pulsatility of the MBR in a healthy left eye, first over four successive heartbeats (Fig. 3a), followed by a single heartbeat on an expanded time scale (Fig. 3b), showing changes in both the CHZ and areas outside the CHZ. The average, maximum, and minimum MBR, as well as pulsatility, were higher outside the CHZ than within it ( *P* < 0.0001 for most indices). Additionally, the time taken to reach the peak MBR was shorter outside the CHZ than within it (*P* = 0.028 for ATI).

### Correlation of average blood flow within the choroidal hypoperfusion zone and outside the choroidal hypoperfusion zone with other factors

Spearman’s correlation analysis of dependent variables revealed that the average MBR in the CHZ was significantly and positively correlated with BS and FAI (Spearman’s *r* = 0.810 and 0.842, respectively; both *P* < 0.0001). Furthermore, the average MBR in the CHZ was significantly and negatively correlated with the vascular resistance and ATI (Spearman’s *r* = -0.920 and − 0.292, respectively; *P* < 0.0001 and *P* = 0.003) (Table 3). The average MBR outside the CHZ was significantly and positively correlated with BS and FAI (Spearman’s *r* = 0.406 and 0.496, respectively; both *P* < 0.0001) and was negatively correlated with vascular resistance (Spearman’s *r* = -0.631; *P* < 0.0001) (Table 3).

**Table 3:** Spearman’s Correlation between Each parameter in the study subjects and the average MBR within and outside the choroidal hypoperfusion zone.

	Average MBR in the CHZ		Average MBR in outside the CHZ	
	Spearman’s r or mean ± SD	*P*-value	Spearman’s r or mean ± SD	*P*-value
Male: Female	4.58 ± 2.17: 3.92 ± 1.57	0.197	9.54 ± 1.18: 9.15 ± 1.41	0.123
Age	−0.293	0.003	−0.136	0.179
Body mass index	−0.103	0.309	−0.034	0.739
Intraocular pressure	−0.020	0.847	−0.073	0.473
Systolic blood pressure	−0.016	0.874	0.026	0.795
Diastolic blood pressure	0.019	0.848	0.059	0.562
Mean blood pressure	−0.016	0.874	0.042	0.678
Ocular perfusion pressure	−0.003	0.973	0.095	0.346
Heart rate	−0.158	0.117	−0.158	0.118
Vascular resistance in the CHZ	−0.920	< 0.0001	−0.631	< 0.0001
Beat strength in the CHZ	0.810	< 0.0001	0.406	< 0.0001
FAI in the CHZ	0.842	< 0.0001	0.496	< 0.0001
ATI in the CHZ	−0.292	0.003	−0.105	0.298
RI in the CHZ	−0.037	0.714	−0.035	0.730
BOS in the CHZ	0.036	0.722	0.037	0.718
BOT in the CHZ	0.123	0.223	−0.061	0.547
Skew in the CHZ	0.155	0.124	0.038	0.705

Data are expressed as the mean ± standard deviation for 100 healthy eyes.

CHZ, Choroidal hypoperfusion zone; MBR, Mean blur rate; CWZ, Choroid watershed zone; FAI, Flow acceleration index; ATI, Acceleration time index; RI, Resistance Index BOS, Blowout score; BOT, Blowout time.

Sex-stratified analysis of CHZ and non-CHZ LSFG parameters revealed no significant sex-related differences in the principal perfusion indices, including average MBR and maximum MBR, within or outside the CHZ in our cohort (Supplementary Table S2). BOS was significantly higher in men in both regions, whereas minimum MBR was significantly higher and ATI was significantly lower in men outside the CHZ.

### Influence of the choroidal hypoperfusion zone on blood flow in sectors of the optic nerve head tissue lying within the choroidal hypoperfusion zone

Table 4 presents the characteristics and average MT (tissue LSFG blood flow within the optic nerve head in between retinal arteriole and venules on the surface of the optic nerve head) of the four ONH quadrants. Age, OPP, and average MT from all ONH quadrants averaged together were not significantly different between the asymmetry and symmetry groups. No significant difference in superior-to-inferior average MT ratio was observed between both groups. In contrast, the temporal-to-nasal average MT ratio was significantly lower in the asymmetry group (0.637 ± 0.137) than in the symmetry group (0.738 ± 0.144; *P* = 0.0015). Although not statistically significant, the average MT in the nasal ONH tended to be higher in the asymmetry group (9.20 ± 2.68) compared to the symmetry group (8.21 ± 2.28; *P* = 0.077).

**Table 4:** Comparison between asymmetry group vs. symmetry group.

	Asymmetry group	Symmetry group	*P*-value
Subjects	67	33	
Age (years)	41.0 ± 13.1	44.6 ± 17.5	0.585
Ocular perfusion pressure (mmHg)	47.9 ± 6.77	47.8 ± 7.62	0.529
Average MT in Superior ONH (AU)	8.39 ± 2.27	8.28 ± 2.18	0.874
Average MT in Temporal ONH (AU)	5.72 ± 1.68	6.05 ± 2.00	0.447
Average MT in Inferior ONH (AU)	8.17 ± 2.16	8.33 ± 2.34	0.803
Average MT in Nasal ONH (AU)	9.20 ± 2.68	8.21 ± 2.28	0.077
Temporal: Nasal average MT ratio	0.64 ± 0.137	0.738 ± 0.144	0.0015
Superior: Inferior average MT ratio	1.04 ± 0.167	1.01 ± 0.148	0.541

Data are expressed as the mean ± standard deviation for 100 healthy eyes.

*P*-values were obtained from Mann-Whitney test.

OPP, ocular perfusion pressure; MT, Mean blur rate in the optic nerve head tissue: ONH, Optic nerve head.

## Discussion

In the present study, we demonstrated that the LSFG color blood flow maps defined areas of lower blood flow constituting the choroidal watershed and peripapillary hypoperfusion zones, and their location showed a strong qualitative spatial correspondence when the color blood flow map averaged over the cardiac cycle was compared to the locations of slowed filling of dye in the early phases of FA in illustrative NAION cases. This finding provides evidence that the CHZ defined by LSFG is related to areas of delayed filling of dye on fluorescein angiography. Additionally, we showed that reduced choroidal blood flow, flow acceleration, and pulsatility were all reduced within the CHZ, indicating the microvascular resistance is likely to be greater in the CHZ. Furthermore, we found that eyes with a symmetric CHZ distribution exhibited considerably delayed and blunted blood flow waveforms—characterized by higher ATI and lower skew—compared to those with asymmetric CHZ patterns, consistent with higher vascular resistance and lower blood flow. Finally, we determined that the location of the CHZ with respect to the optic nerve head corresponded to the sectors of lower ONH tissue blood flow that lie within the hypoperfusion zone, most likely due to the shared blood supply from branches of the posterior ciliary arteries.

The area of CHZ is dynamic and expands during the diastolic phase and contracts during the systolic phase. During diastole the center of the CHZ shows the lowest blood flow and tissue within this location would be expected to be at the greatest risk of ischemia during diastole and when ocular perfusion pressure falls below a critical level. Previous studies using FA and IA showed that well-outlined watershed zones were observed in 40%–45% of healthy participants^8,9^. However, determining CHZ using these techniques remains challenging, as it is only detectable in the early phase and exhibits rapid fluorescein dye filling and may not be captured considering the digital sampling rate of ocular angiography, especially in eyes with higher ocular perfusion pressure and blood flow at the time of the angiogram. LSFG on the other hand provides temporal changes in blood flow through each cardiac cycle making delineation of spatial patterns of hypoperfusion more easily discernable. Using LSFG, it appears that CHZs are present in every eye, but their location and extent vary depending upon the spatial distribution of choroidal vessels that occurs during development for each eye and in each individual. In addition, the end artery vascular resistance and ocular perfusion pressure will influence the size and extent of hypoperfusion at any given time. Although fluorescein and indocyanine green angiography provide visualization of the CHZ in most eyes^8,10,27^. it cannot effectively show differences in CHZ area between systolic and diastolic phases. In contrast, LSFG may be useful for readily providing information on CHZ area during both systolic and diastolic phases. Watanabe et al. demonstrated the peripapillary CHZ in eyes with polypoidal choroidal vasculopathy using LSFG^29^. However, they only examined small, narrow angles of view of the LSFG composite flow map and angiography. Therefore, we created a panoramic LSFG color composite map and blood flow maps during the systolic and diastolic phases to identify the correspondence of the posterior and peripheral CHZs between the LSFG and angiography. Considering these findings, along with LSFG’s non-invasive nature and lack of need for injected contrast agents, LSFG is positioned as a powerful tool for assessing the dynamic nature of the CHZ in both healthy and diseased eyes.

This study is the first to demonstrate reduced blood flow and pulsatility in CHZ due to higher vascular resistance. Furthermore, low flow acceleration and delayed time to peak MBR were observed in the CHZ of the 100 healthy eyes. Generally, the CHZ exists between the distribution territories of any two ends PCAs, with the primary direction of blood flow in the distal short PCAs directed toward the equator. This orientation results in lower blood pressure in their small branches supplying the peripapillary choroid, rendering it a low-perfusion system compared to the rest of the main choroid^3^. Regarding the relationship between pulsatile flow and vascular resistance, Nakano et al. have shown that peripheral vascular resistance was markedly lower in pulsatile flow than in non-pulsatile flow in their animal study. This may be attributed to the release of nitric oxide from endothelium, which is induced by pulsatile flow^30^. In the present study, the average MBR in both the CHZ and outside the CHZ was positively correlated with BS and FAI, and it was negatively correlated with vascular resistance. Considering the direction and streaming blood flow of PCAs and the findings mentioned above, it is reasonable to conclude that the CHZ’s reduction in blood flow, flow acceleration, and pulsatility are attributed to increased vascular resistance. Importantly, while the correlations between average MBR and BS/FAI are strong within the CHZ (Spearman’s *r* = 0.810 and 0.842), they are considerably weaker outside the CHZ (*r* = 0.406 and 0.496), suggesting that the CHZ microvascular bed has a qualitatively different physiological relationship between perfusion magnitude and waveform characteristics. BS reflects the amplitude of the pulsatile flow waveform; a lower BS in the CHZ indicates not merely that average flow is lower, but that the cardiac-cycle-driven pulsatile drive is blunted — consistent with elevated distal vascular resistance. Similarly, FAI measures the rate of systolic flow acceleration, a parameter governed by proximal vessel compliance and resistance; its reduction in the CHZ is consistent with a high-resistance, low-compliance microvascular territory that cannot rapidly accelerate flow during systole. These findings therefore represent physiologically meaningful alterations in vascular hemodynamics beyond what would be expected from reduced average flow alone. The delayed ATI — reflecting prolonged time-to-peak systolic flow — further supports the interpretation that the CHZ represents a region of true hemodynamic disadvantage, as this timing parameter is not mathematically derivable from average MBR magnitude. This finding suggests that this vascular bed is inherently less capable of responding to perfusion challenges.

Regarding the relationship between the location of the CHZ and the corresponding sectors of ONH tissue blood flow, we demonstrated that the temporal-to-nasal average MT ratio was significantly lower in the asymmetry group than in the symmetry group. In contrast, no significant difference in the superior-to-inferior average MT ratio was observed between the asymmetry and symmetry groups. Consistent with previous reports indicating that the most typical locations of the CHZ (60%) include the temporal part of the ONH and the adjacent peripapillary choroid^1^, all 67 eyes in the asymmetry group (67% of all 100 participants) in this study showed that the CHZ existed only on the temporal side of the ONH. Hayreh previously reported that in eyes with AION and glaucomatous optic neuropathy, the location of the watershed zone influences the susceptibility of the corresponding ONH region to ischemia during a reduction in perfusion pressure in the PCAs or their branches^1,31^. Additionally, Sato et al. reported that the mean deviation of the visual field in a patient with normal-tension glaucoma (NTG) was greater in eyes where the CHZ encompassed a larger portion of the ONH than in the opposite eye^4^. They concluded that the location of the watershed zone might influence the progression of visual field defects in patients with NTG^4^. Considering these findings and that the lamina cribrosa and the prelaminar region are also supplied by distal branches arising directly from the short PCAs^1^, the specific location of the CHZ relative to the ONH, along with its shared blood supply with the prelaminar optic nerve, may heighten the risk of ischemic disorders affecting the nerve sectors within it.

Our findings are consistent with the notion that impaired ocular perfusion and autoregulation contribute to focal ischemic vulnerability. Notably, Park et al. recently reported that excessive nocturnal blood pressure dipping was associated with rapid central visual field loss in normal-tension glaucoma^32^, suggesting that transient nocturnal hypoperfusion may exacerbate damage in regions corresponding to the CWZ. The reduced flow and delayed pulsatility observed in our study may thus represent structural correlates of this functional vulnerability.

The intergroup comparison between symmetry and asymmetry types revealed that ATI was significantly higher, and skew was lower in the symmetry group, suggesting delayed and blunted blood flow waveforms. These findings indicate that eyes with a more extensive CHZ may experience greater hemodynamic stress, even in the absence of overt disease and may be associated with greater risk of ischemia of the tissues lying within it. The spatial extent and waveform characteristics of the CHZ may therefore influence susceptibility to ischemic events and could serve as a non-invasive biomarker for identifying individuals at higher risk for ischemic optic nerve damage.

The present study has some limitations. First, we investigated only the left eye of healthy participants. Although a subgroup analysis of 45 individuals with bilateral LSFG data revealed no significant interocular differences in choroidal blood flow or pulse waveform parameters (see Supplementary Table S1), previous studies have reported subtle physiological laterality in ONH microcirculation among healthy subjects using LSFG^33^. We think that it is essential to examine the differences in the presence of the CWZ and MBR between the right and left eyes, especially in patients exhibiting inter-eye asymmetry of chronic progressive damage to the optic nerve as in some patients with asymmetric glaucoma. Further studies are required to evaluate whether there are substantial differences in these CHZ parameters between the right and left eyes of patients with asymmetric glaucoma.

Second, the LSFG analyzer was unable to differentiate blood flow between the choriocapillaris and large choroidal vessels in the Sattler’s and Haller’s layers. Although we placed the rubber band in the choroidal regions while avoiding the large choroidal vessels when they could be visualized when we analyzed choroidal blood flow, MBRs in the choroidal regions may have included blood flow in both layers of the choriocapillaris and large choroidal vessels. Third, axial eye length and refractive error were not measured in this study. These variables are potentially important for two reasons: (1) Axial length affects the lateral magnification of LSFG images, which influences the apparent spatial extent of the CHZ and the dimensions of the rubber band ROIs; eyes with greater axial length have higher image magnification, potentially affecting the area sampled. (2) High myopia is associated with structural remodeling of the peripapillary choroid, including thinning and altered vascular architecture, which may change the spatial distribution and size of the CHZ^34^. Our cohort was not screened for high myopia, and we cannot exclude that some participants had long axial lengths that influenced their CHZ pattern. Future studies should include axial length and refraction measurements, with high myopes either excluded or analyzed separately. Further studies are required to assess whether the refractive state and retinal thinning in high myopia affects the location of the choroidal watershed and hypoperfusion zone which could impart additional risk of ischemic damage. Fourth, this study defined the region of interest within a CHZ as the area within contiguous pixels having the lowest blood flow that were plausible zones between end artery posterior ciliary artery distributions as previously defined with fluorescein and indocyanine green angiography. Future studies could employ a more objective, threshold-based segmentation (e.g., contiguous pixels having low blood flow below a defined MBR percentile, such as the 20th percentile, for example) to improve reproducibility. Additionally, a key limitation of our statistical approach is that a correction for multiple comparisons was not applied; therefore, findings for secondary waveform parameters should be considered exploratory. In addition, while sex differences in systemic hemodynamics were noted, sex was not included as a covariate in the statistical models.

A sex-stratified analysis of CHZ and non-CHZ LSFG parameters was performed as an additional analysis, and the results are reported in Supplementary Table S2. Consistent with prior published reports demonstrating that sex differences in LSFG parameters are present for ONH blood flow but not clearly observed for choroidal MBR^28^, no significant sex-related differences were observed in the principal perfusion indices, including average MBR and maximum MBR, within or outside the CHZ in our cohort. However, BOS was significantly higher in men in both regions, whereas minimum MBR was significantly higher and ATI was significantly lower in men outside the CHZ. These differences may partly reflect differences in systemic and ocular perfusion status, because men in our cohort also had significantly higher DBP and ocular perfusion pressure than women.

Fifth, the mean BMI of the cohort was approximately 28 kg/m² in both males and females, which is slightly above the normal range. It is possible that findings may not be fully generalizable to lean individuals, and future studies in cohorts stratified by BMI would be informative. Sixth, choroidal thickness was not assessed by OCT in this study. Recent unpublished work from our group in normal eyes and in eyes with AMD has demonstrated that choroidal thickness measured by OCT is poorly correlated with choroidal blood flow measured by LSFG, suggesting that thickness and perfusion represent complementary rather than redundant measures of choroidal health; nevertheless, the absence of thickness data limits the ability to determine whether structural choroidal differences contribute to the hemodynamic patterns observed. Seventh, this was an exploratory, hypothesis-generating study; no formal a priori power calculation was performed. The sample size of 100 healthy eyes was chosen to provide sufficient representation across a range of CHZ spatial patterns (asymmetric vs. symmetric) and to enable subgroup comparisons. The highly significant results obtained for the primary comparisons (*P* < 0.0001 for all key flow metrics) suggest adequate statistical power for the main study endpoints. Sex-stratified analyses of choroidal MBR and waveform parameters within and outside the CHZ were performed to address this limitation; no significant sex differences in choroidal LSFG parameters were observed (see Supplementary Table S2), consistent with prior reports demonstrating that sex-related differences in LSFG are confined to the optic nerve head and are absent in the choroidal circulation^28^.

The original contributions of this study are as follows: (1) This is the first quantitative characterization of pulsatile waveform parameters within the CHZ, including ATI, FAI, BS, and RI — parameters that directly reflect microvascular hemodynamic resistance and compliance and could not be assessed by prior angiographic methods. (2) This is the first demonstration of delayed time-to-peak systolic blood flow within the CHZ, indicating not merely lower average perfusion but a distinct temporal pattern of pressure propagation through this microvascular territory that has direct implications for understanding ischemic vulnerability. (3) This is the first characterization of the dynamic, cardiac-cycle-dependent behavior of the CHZ, showing that the zone expands during diastole and contracts during systole — a finding that explains why transient nocturnal hypotension and drops in ocular perfusion pressure are particularly injurious to ONH tissue within the watershed zone. (4) This is the first demonstration that the spatial pattern of the CHZ relative to the ONH (asymmetric vs. symmetric) predicts the temporal-to-nasal ONH blood flow ratio, providing a mechanistic link between watershed zone anatomy and differential sectoral vulnerability of the ONH to ischemia. (5) This study provides the first validation of LSFG as a non-invasive, contrast-free method for visualizing the CHZ, demonstrating qualitative spatial correspondence to the areas of delayed choroidal filling on fluorescein angiography in illustrative NAION cases.

In summary, this study demonstrated that blood flow, pulsatility, and flow acceleration are significantly reduced in the choroidal watershed and peripapillary choroidal hypoperfusion zones due to higher vascular resistance, making its vascular bed more vulnerable to dips in OPP from either arterial hypotension or a spike in intraocular pressure. It is known that some patients are more susceptible to optic nerve damage when this occurs, especially during sleep and during anesthesia or shock^35–43^. The spatial location of the choroidal watershed and hypoperfusion zones relative to the ONH and its shared blood supply to the prelaminar optic nerve may pose an additional risk to the nerve sectors lying within it in ischemic disorders of the optic nerve. The findings underscore the utility of LSFG in non-invasively assessing choroidal blood flow, providing a valuable tool for clinicians in diagnosing and monitoring ischemic disorders affecting the optic nerve.

## Methods

### Participants

We studied 100 left eyes of 100 healthy participants (43 male and 57 females, mean age 42.2 ± 14.7 years). Additionally, two eyes of two patients with NAION were reviewed as illustrative cases to demonstrate the qualitative correspondence with FA findings. Blood flow dynamics were quantified using LSFG-NAVI (LSFG-NAVI device, Softcare Co. Ltd, Fukuoka, Japan). All healthy participants underwent an ophthalmic examination, slit-lamp examination, optical coherence tomography, and scanning laser ophthalmoscopy examination to evaluate whether their eyes were normal. Inclusion criteria were: age 18 years or older, best-corrected visual acuity of 20/25 or better, normal optic disc appearance on clinical examination and scanning laser ophthalmoscopy, and no structural abnormalities detected on OCT of the optic nerve head or peripapillary retina. Participants were excluded if they had a medical history of systemic diseases, such as diabetes, hypertension, hyperlipidemia, ocular disease, treatment with any medication, smoking habits, or were pregnant.

Among these 100 eyes, 45 participants (17 males and 28 females, mean age 49.9 ± 15.3 years) had available bilateral LSFG measurements. To determine whether analysis could be limited to one eye, we compared LSFG parameters between right and left eyes in this subgroup. As no significant interocular differences in choroidal blood flow or pulse waveform parameters were observed (see Supplementary Table S1), we selected only the left eye for standardization of data analysis. A formal analysis of inter-ocular symmetry in the spatial patterns of the CHZ was not performed. In the two eyes with NAION, FA was used to spatially correlate the location of the CWZ and peripapillary choroidal hypoperfusion zone observed during the early phase of fluorescein dye filling of the choroid with a panoramic montage composed of LSFG color blood flow maps.

This study adhered to the tenets of the Declaration of Helsinki and was approved by the Institutional Review Board of the University of Iowa (IRB number: 201801713). Written informed consent was obtained from all participants after explaining the nature and possible consequences of the study.

### LSFG measurement, analysis of blood flow, pulse waveforms and beat strength

LSFG consists of a retinal imager with a maximum 21°field of view, equipped with an 830-nm diode laser as the light source and a standard charge-coupled device sensor (750 width × 360 height pixels) as the detector^11,12^. A dynamic speckle pattern is produced and imaged in the plane of the retina. Erythrocyte movement interferes with light caused by scattering and blurs the speckle pattern at each pixel in proportion to the velocity of the red blood cell movement. The blur rate of each pixel can be quantified by analyzing each video frame in series. The MBR measures relative blood flow velocity, expressed in arbitrary units (AU), and is determined by analyzing the speckle contrast pattern produced by the moving erythrocytes in the ocular tissue^12^. The MBR images are acquired at a rate of 30 frames/s over a 4-second period, capturing approximately four heart cycles. Participants were instructed to fixate on an internal target light.

Using LSFG, the mean blur rate, which is proportional to red blood cell velocity at each pixel, is displayed as a color blood flow map at each of the time points sampled during the cardiac cycle (30 per second). An average color blood flow map is displayed which shows the color-coded blood flow at each pixel averaged over 4 cardiac cycles. In addition, in a subset of eyes, a number of adjacent, overlapping regions around the optic nerve and macula were imaged in rapid succession and combined to create a panoramic montage blood flow map so that the spatial distribution of the choroidal hypoperfusion zones could be discerned over a larger fundus area. This montage was created by manually aligning and stitching the individual images, as this is not an automated feature of the software.

### LSFG image analysis: definition of choroidal hypoperfusion zone and quantification of blood flow

LSFG Analyzer software (version 3.5.0.0; Softcare) was used to quantify blood flow within user-defined regions of interest (ROIs). In this software, ROIs are termed “rubber bands,” and LSFG analysis currently requires the manual placement of these polygonal ROIs to measure blood flow at desired locations.

**Fig. 4:**
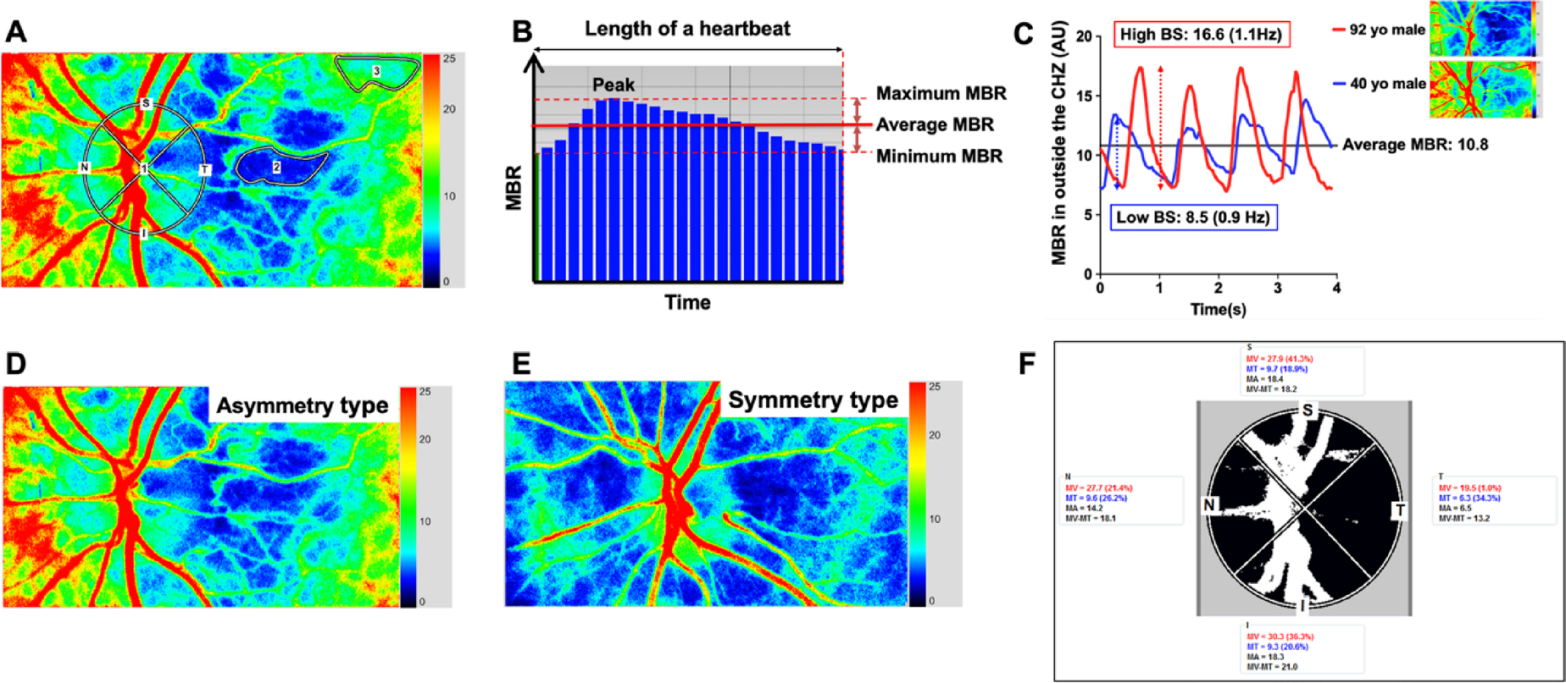
Analysis of the choroidal hypoperfusion zone (CHZ) and optic nerve head (ONH) using the LSFG analyzer. (**A**) The margin of the CHZ (inner boundary), outside the CHZ (outer boundary) are manually delineated using spline-based regions of interest (“rubber bands”). The ONH margin was identified using a spline and elliptical boundary in a laser speckle flowgraphy (LSFG) analyzer. (**B**) LSFG analyzer can provide the minimum, average, and maximum mean blur rate (MBR) from the defined regions over an averaged cardiac cycle. (**C**) The beat strength (BS) is calculated based on the change of MBR over the measurement time and is proportional to the amplitude between the maximum and minimum MBR, reflecting blood flow. This figure shows four heat beats of a 90-year-old man (Red color) and a 40-year-old man (blue color). While both participants had the same average MBR value of 10.8, BS was higher in a 90-year-old male (16.6) than in a 40-year-old male (8.5). (**D**) An example of the Asymmetry type, where the CHZ is located solely on the temporal side of the ONH. (**E**) An example of the symmetry type, where the CHZ encompasses the entire ONH. (**F**) The analyzer automatically distinguishes between ONH vessels (MV) depicted as white pixels and tissue (MT) depicted as black pixels and can analyze MT in four quadrants.

In this study, we defined the LSFG-visible peripapillary choroidal hypoperfusion zone, which corresponds to part of the CWZ, as the ‘choroidal hypoperfusion zone’ (CHZ). To clarify terminology used throughout this manuscript: CWZ refers to the anatomically defined choroidal watershed zone — the inter-arterial boundary territory between adjacent ciliary artery perfusion domains, as originally described by Hayreh. CHZ refers specifically to the LSFG-visible peripapillary choroidal hypoperfusion zone, representing the functional blood-flow correlate of the CWZ as detected by LSFG. While these zones correspond spatially, they are conceptually distinct: the CWZ is an anatomical territory defined by vascular supply boundaries, whereas the CHZ is operationally defined by measured blood flow reduction on LSFG color maps. The CHZ was identified visually as the area of lowest blood flow, appearing as the region of cool, blue color on the LSFG composite color map (Fig. 4a). This visual identification is qualitative, as a specific MBR threshold was not used to define the CHZ boundary. This approach is consistent with the conventional identification of the choroidal watershed zone on fluorescein and indocyanine green angiography, where the zone is operationally defined as the region of lowest perfusion between adjacent ciliary artery territories. Importantly, the primary novel quantitative findings of this study — the waveform timing parameters (ATI, FAI), the dynamic diastolic expansion of the CHZ, and the relationship between CHZ spatial pattern and sectoral ONH blood flow, do not follow necessarily from this definition, and therefore are not subject to circularity. The lower average MBR within the CHZ, while partly definitional, serves as an internal validation that the visually-identified zone corresponds to a physiologically distinct microvascular territory. Based on this definition, two distinct ROIs were placed for analysis: one within the blue-colored CHZ and an adjacent, well-perfused area (warm, yellow-to-red colors) outside the CHZ, while carefully avoiding major retinal and choroidal vessels (Fig. 4a). To ensure consistency, all ROIs were placed by a single trained operator (R.H.). A formal assessment of inter- or intra-observer reproducibility was not performed, which is a limitation of this study. Similarly, classification of eyes as asymmetric or symmetric CHZ pattern was performed by a single trained examiner (R.H.) without independent second-observer verification; formal inter-rater reliability assessment was not conducted, which represents an additional limitation. Once the ROIs were defined, the LSFG analyzer calculated the minimum, average, and maximum MBR for each region (Fig. 4b). The minimum MBR was defined by the lowest MBR value, the average MBR by the mean MBR value, and the maximum MBR by the peak MBR during one average cardiac cycle from the four cycles recorded. The hemodynamic pattern observed in the LSFG mainly originates from the choroid when the major retinal vessels are excluded from the measurement site^20^. In LSFG blood flow studies using 830 nm wavelength, the choroidal blood flow dominates the blood flow in areas between retinal arterioles and venules^44^. Therefore, the LSFG provides quantitative changes in the choroidal circulation of an imaged eye. In addition to the MBR analysis, the LSFG analyzer provides various parameters characterizing the shape of the pulsatile flow waveform during the cardiac cycle. In this study, FAI, ATI, BOS, BOT, RI, and skew were analyzed as the parameters of the pulsatile waveforms (see Supplementary Fig. S1).

### Beat strength (BS) as a new parameter in LSFG analysis

The BS is calculated based on the change in MBR over the measurement time and is proportional to the amplitude between the maximum and minimum MBR as blood flow, reflecting the magnitude of pulsatile flow during each heartbeat (Fig. 4c). A higher BS indicates greater fluctuations in blood flow during the cardiac cycle, that may be influenced by peripheral vascular resistance, vascular stiffness, difference between systolic and diastolic pressure and cardiac stroke volume. In addition, BS is defined by the power of the frequency at which the maximum spectral power was observed in the laser speckle flow signal. The power spectrum was derived from the frequency distribution of MBR values, considering only frequencies higher than 0.5 Hz. This approach enables quantification of pulsatile blood flow fluctuations during one cardiac cycle. The formula used to calculate BS has been disclosed in an international patent application (PCT/JP2016/084746) and applied in previous LSFG studies^33,45^.

### Comparison of optic nerve head blood flow in eyes with symmetric vs. asymmetric location of choroidal hypoperfusion zone with respect to the center of the optic nerve head

To investigate the influence of CHZ on adjacent ONH tissue blood flow, we classified all 100 eyes into asymmetry and symmetry groups.

We defined the asymmetry type based on the LSFG color flow map relative to the ONH. In these cases, only the temporal portion of the optic nerve was located within the CHZ (Fig. 4d) creating an asymmetry of blood flow between the temporal and nasal portions of the optic nerve head. The symmetry type showed a symmetrical color pattern between the nasal and temporal sides of the ONH, indicating that the entire optic nerve was located within the CHZ (Fig. 4e). This visual classification is a novel approach for this study, developed to functionally assess the impact of the CHZ’s location relative to the ONH.

We identified the margin of the ONH using a circular or elliptical boundary to analyze ONH tissue blood flow (Fig. 4f). ONH blood flow can be automatically divided into MBR from retinal arterioles and venules coursing along the superficial optic nerve head and MBR from intervening capillary tissue between the retinal arterioles and venules using a definitive automated thresholding algorithm provided by the LSFG Analyzer software (Ver. 3.5.5.0, Softcare, Ltd.). The MBR in the ONH tissue (Mean blur rate in the ONH tissue, MT) has shown to be linearly correlated with the deep ONH capillary blood flow from the prelaminar and lamina cribrosa regions, regardless of fundus pigmentation^46^. This finding is supported by animal study in which MT was highly correlated with ONH capillary blood flow measured by the hydrogen gas clearance method, validating the use of MT for interindividual and intergroup comparisons in the current study^46^. The average MT values in each quadrant (i.e., superior, inferior, nasal, and temporal), which are automatically segmented by the software, were used to calculate the temporal-to-nasal and superior-to-inferior MT ratios in the ONH tissue (Fig. 4f)^47^. These ratios were then compared between the asymmetry and symmetry groups to assess quadrant-level distribution imbalance.

In addition, we compared choroidal blood flow and pulsatile waveform parameters within and outside the CHZ between the asymmetry and symmetry groups. This included average, maximum, and minimum MBR, vascular resistance, and pulse waveform indices such as BS, FAI, ATI, RI, BOT, BOS, and skew.

### Vascular resistance

Vascular resistance was determined using the formula Vascular resistance = OPP/blood flow^48^. We used the OPP/MBR AU as an indicator of vascular resistance (in arbitrary units) and calculated it in choroidal vascular beds in the present study.

Ocular Hemodynamics: Calculation of Ocular Perfusion Pressure.

We measured IOP and BP at the time of LSFG recording to calculate OPP. We calculated the mean blood pressure (MBP) from SBP and DBP using the following equation: MBP = 1/3 (SBP – DBP) + DBP^49^. The equation used to calculate OPP was as follows: OPP (sitting position) = 2/3 MBP – IOP^49^.

### Sex related differences in blood flow

A sex-stratified analysis of CHZ and non-CHZ LSFG parameters was performed as an additional analysis.

### Statistical analysis

All data are expressed as the mean ± standard deviation (SD). The Shapiro–Wilk test was used to assess the normality of the distributions. We used the Mann–Whitney U test and Wilcoxon test to compare the participants’ backgrounds and LSFG parameters. Spearman’s correlation coefficients were used to assess the relationships between the variables. The statistical significance level was set at *P* < 0.05. All statistical analyses were performed using GraphPad Prism version 8.0.1 macOS (GraphPad Software, San Diego, CA, USA).

## Supplementary Information

Below is the link to the electronic supplementary material.

Supplementary Material 1

Supplementary Material 2

## Data Availability

The data that support the findings of this study are available from the corresponding author, upon reasonable request.
