# Supplementary figures and images for: Laser speckle flowgraph reveals dynamic characteristics and clinical relevance of choroidal watershed and peripapillary hypoperfusion zones
Source: Sci Rep. 2026 Apr 4;16:16251. doi: 10.1038/s41598-026-47062-z (PMC13201649; doi:10.1038/s41598-026-47062-z)

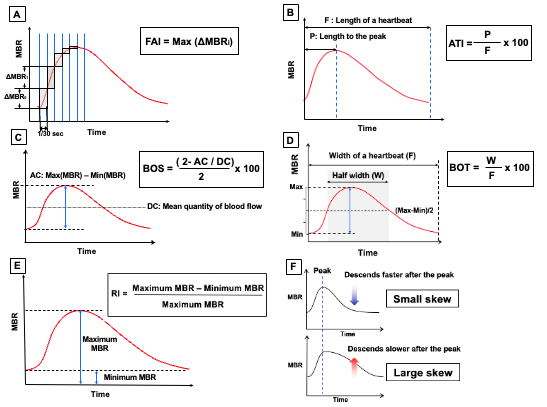

Supplement: Supplementary file 1 — Supplementary Material 1 [file 41598_2026_47062_MOESM1_ESM.tiff]
