# Supplementary material for: Laser speckle flowgraph reveals dynamic characteristics and clinical relevance of choroidal watershed and peripapillary hypoperfusion zones
Source: Sci Rep. 2026 Apr 4;16:16251. doi: 10.1038/s41598-026-47062-z (PMC13201649; doi:10.1038/s41598-026-47062-z)
Supplement: Supplementary file 2 — Supplementary Material 2 [file 41598_2026_47062_MOESM2_ESM.docx]

**Laser Speckle Flowgraphy Reveals Dynamic Characteristics and Clinical Relevance of Choroidal Watershed and Peripapillary Hypoperfusion Zones**

**Supplementary Table S1. Comparison of LSFG Parameters Between Right and Left Eyes Within and Outside the Choroidal Hypoperfusion Zone in 45 Participants**

|  | **Right Eye in the CHZ**  **(n = 45)** | **Left Eye in the CHZ. (n = 45)** | **P-value** | **Right Eye outside the CHZ (n = 45)** | **Left Eye outside the CHZ (n = 45)** | **P-value** |
| --- | --- | --- | --- | --- | --- | --- |
| **Average MBR (AU)** | 3.88 ± 1.54 | 4.00 ± 2.05 | 0.639 | 9.68 ± 2.45 | 9.21 ± 1.54 | 0.475 |
| **Maximum MBR (AU)** | 5.01 ± 1.91 | 5.19 ± 2.53 | 0.769 | 12.5 ± 3.29 | 11.9 ± 1.92 | 0.493 |
| **Minimum MBR (AU)** | 2.98 ± 1.26 | 3.05 ± 1.62 | 0.504 | 7.45 ± 2.11 | 7.07 ± 1.37 | 0.309 |
| **Vascular Resistance** | 14.5 ± 6.26 | 15.0 ± 7.43 | 0.911 | 5.33 ± 1.55 | 5.39 ± 1.20 | 0.192 |
| **Beat Strength** | 3.65 ± 1.63 | 3.71 ± 1.74 | 0.617 | 8.94 ± 3.03 | 8.16 ± 2.08 | 0.193 |
| **FAI** | 0.61 ± 0.27 | 0.64 ± 0.34 | 0.894 | 1.59 ± 0.77 | 1.48 ± 0.39 | 0.760 |
| **ATI** | 29.6 ± 6.09 | 29.8 ± 5.31 | 0.971 | 29.6 ± 5.65 | 28.8 ± 5.66 | 0.455 |
| **RI** | 0.41 ± 0.08 | 0.42 ± 0.08 | 0.183 | 0.41 ± 0.09 | 0.40 ± 0.07 | 0.879 |
| **BOS** | 72.4 ± 6.99 | 71.4 ± 7.45 | 0.153 | 72.3 ± 7.92 | 72.7 ± 5.95 | 0.979 |
| **BOT** | 47.8 ± 6.26 | 46.7 ± 5.53 | 0.314 | 47.2 ± 6.20 | 47.8 ± 4.53 | 0.479 |
| **Skew** | 13.2 ± 2.93 | 13.6 ± 2.19 | 0.918 | 13.0 ± 2.81 | 13.3 ± 2.13 | 0.609 |

Data are expressed as the mean ± standard error.

P-values were obtained from Wilcoxon test.

CHZ, Choroidal hypoperfusion zone; MBR, Mean blur rate; Beat strength, BS; FAI, Flow acceleration index; ATI. Acceleration time index; RI, Resistance index; BOS, Blowout score; BOT, Blowout time.

**Supplementary Table 2. Comparison of LSFG Parameters and Vascular Resistance Between Male and Female within and outside the Choroidal Hypoperfusion Zone**

|  | **CHZ** | | | **Outside the CHZ** | | |
| --- | --- | --- | --- | --- | --- | --- |
|  | **Male**  **(n = 43)** | **Female**  **(n = 57)** | **P-value** | **Male**  **(n = 43)** | **Female**  **(n = 57)** | **P-value** |
| **Average MBR (AU)** | 4.58 ± 2.16 | 3.92 ± 1.57 | 0.197 | 9.54 ± 1.18 | 9.15 ± 1.41 | 0.123 |
| **Maximum MBR (AU)** | 5.86 ± 2.71 | 5.13 ± 2.03 | 0.294 | 12.0 ± 1.75 | 11.8 ± 1.77 | 0.476 |
| **Minimum MBR (AU)** | 3.56 ± 1.72 | 2.95 ± 1.20 | 0.106 | 7.49 ± 1.90 | 7.04 ± 1.30 | 0.017 |
| **Beat Strength** | 3.97 ± 1.89 | 3.91 ± 1.70 | 0.990 | 7.82 ± 2.36 | 8.19 ± 1.95 | 0.362 |
| **FAI** | 0.76 ± 0.41 | 0.65 ± 0.31 | 0.272 | 1.50 ± 0.45 | 1.45 ± 0.39 | 0.519 |
| **ATI** | 26.8 ± 5.66 | 28.8 ± 4.92 | 0.224 | 26.5 ± 4.51 | 28.4 ± 5.61 | 0.006 |
| **RI** | 0.39 ± 0.07 | 0.42 ± 0.06 | 0.072 | 0.38 ± 0.07 | 0.40 ± 0.07 | 0.055 |
| **BOS** | 73.4 ± 6.70 | 71.1 ± 5.67 | 0.012 | 75.4 ± 5.46 | 72.9 ± 5.73 | 0.034 |
| **BOT** | 48.2 ± 5.34 | 47.3 ± 4.74 | 0.500 | 48.4 ± 6.09 | 47.8 ± 5.09 | 0.327 |
| **Skew** | 13.6 ± 2.24 | 13.6 ± 2.07 | 0.883 | 13.5 ± 2.19 | 13.1 ± 2.11 | 0.927 |

Data are expressed as the mean ± standard error.

P-values were obtained from the Mann-Whitney U test

CHZ, choroidal hypoperfusion zone; MBR, mean blur rate; BS, beat strength; FAI, flow acceleration index; ATI, acceleration time index; RI, resistance index; BOS, blowout score; BOT, blowout time; AU, arbitrary units.

**Supplementary Figure S1. Calculation of the LSFG Parameters of Pulse Waveforms**

**
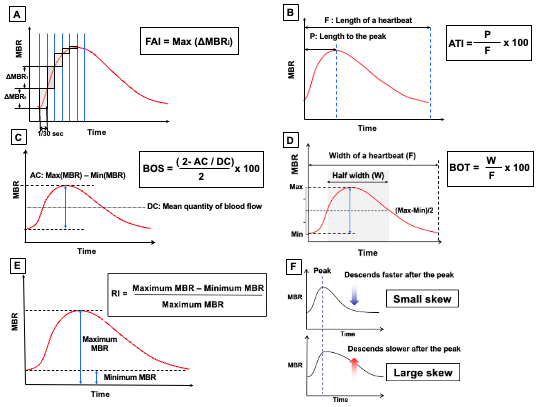
**

**(A)** Flow acceleration index (FAI) represents the maximum rate of change in the MBR, indicating the momentary force that rapidly increases blood flow.

**(B**) Acceleration time index (ATI) is derived from the ratio of the time taken before reaching the peak MBR to the duration of the heartbeat.

**(C)**Blowout score is an indicator of the strength of the blood flow sustained in the vessel between heartbeats. BOS is calculated from the difference between the maximum and minimum MBR.

**(D)** Blowout time (BOT) represents the half-width of the blood flow wave and is the ratio of this half-width duration to one heartbeat.

**(E)** Resistivity index is calculated by dividing the difference between the maximum and minimum MBR by the maximum MBR.

**(F)** High skew decreases more slowly after the peak, and the small skew decreases faster after the peak.
